# An Outside-In Switch in Integrin Signaling Caused by Chemical and Mechanical Signals in Reactive Astrocytes

**DOI:** 10.3389/fcell.2021.712627

**Published:** 2021-08-23

**Authors:** Leonardo A. Pérez, Aysha Rashid, J. Dale Combs, Pascal Schneider, Andrés Rodríguez, Khalid Salaita, Lisette Leyton

**Affiliations:** ^1^Cellular Communication Laboratory, Program of Cellular and Molecular Biology, Center for Studies on Exercise, Metabolism and Cancer (CEMC), Facultad de Medicina, Instituto de Ciencias Biomédicas, Universidad de Chile, Santiago, Chile; ^2^Advanced Center for Chronic Diseases (ACCDiS), Faculty of Chemical and Pharmaceutical Sciences, Faculty of Medicine, Universidad de Chile, Santiago, Chile; ^3^Chemistry Department, Emory University, Atlanta, GA, United States; ^4^Department of Biochemistry, University of Lausanne, Epalinges, Switzerland; ^5^Group of Research and Innovation in Vascular Health, Machine Learning Applied to Biomedicine Group, Vascular Physiology Laboratory, Faculty of Sciences, Universidad del Bío-Bío, Chillán, Chile

**Keywords:** integrin, mechanotransduction, astrocyte, Thy-1 (CD90), cell contractility, astrogliosis

## Abstract

Astrocyte reactivity is associated with poor repair capacity after injury to the brain, where chemical and physical changes occur in the damaged zone. Astrocyte surface proteins, such as integrins, are upregulated, and the release of pro-inflammatory molecules and extracellular matrix (ECM) proteins upon damage generate a stiffer matrix. Integrins play an important role in triggering a reactive phenotype in astrocytes, and we have reported that α_*V*_β_3_ Integrin binds to the Thy-1 (CD90) neuronal glycoprotein, increasing astrocyte contractility and motility. Alternatively, α_*V*_β_3_ Integrin senses mechanical forces generated by the increased ECM stiffness. Until now, the association between the α_*V*_β_3_ Integrin mechanoreceptor response in astrocytes and changes in their reactive phenotype is unclear. To study the response to combined chemical and mechanical stress, astrocytes were stimulated with Thy-1-Protein A-coated magnetic beads and exposed to a magnetic field to generate mechanical tension. We evaluated the effect of such stimulation on cell adhesion and contraction. We also assessed traction forces and their effect on cell morphology, and integrin surface expression. Mechanical stress accelerated the response of astrocytes to Thy-1 engagement of integrin receptors, resulting in cell adhesion and contraction. Astrocyte contraction then exerted traction forces onto the ECM, inducing faster cell contractility and higher traction forces than Thy-1 alone. Therefore, cell-extrinsic chemical and mechanical signals regulate in an outside-in manner, astrocyte reactivity by inducing integrin upregulation, ligation, and signaling events that promote cell contraction. These changes in turn generate cell-intrinsic signals that increase traction forces exerted onto the ECM (inside-out). This study reveals α_*V*_β_3_ Integrin mechanoreceptor as a novel target to regulate the harmful effects of reactive astrocytes in neuronal healing.

## Introduction

Integrins are receptors that span the plasma membrane and anchor cells to the external environment. They transmit signals in and out of the cells, allowing a fluid communication between the extracellular matrix (ECM) and the cytoskeleton. The crosstalk between these extra- and intra-cellular signals transduced by the integrin receptors occurs in both ways, in a process canonically described as outside-in and inside-out signaling. Integrins are initially inactive and thus bind to their ligands with low affinity. Ligand-induced clustering is well known to activate integrins, increasing the affinity and avidity of the interactions and signaling (outside-in) (Oria et al., 2017). Molecular changes occurring intracellularly also assist integrin activation by promoting the formation of integrin-based adhesion complexes (known as focal adhesions), and cytoskeletal changes that convey mechanical stress signals back to the integrins and the ECM (inside-out) (Mohammed et al., 2019).

Integrins also possess mechanosensing properties that allow them to detect rigidity changes of the ECM. Furthermore, a stiff ECM limits lateral integrin diffusion and promotes integrin clustering (Paszek et al., 2014). Therefore, the mechanical stress is transmitted to and from the cells, with integrins as mechanotransducers at focal adhesions. At these focal contacts, rigidity sensing by mechanoreceptors serves to transmit outside-in and inside-out tensional signals (Mohammed et al., 2019). Therefore, contractility generated by ligand-engaged integrins exerts traction forces that are transmitted through these focal points to the ECM by the recruitment of integrins. This means that in a complex environment, integrins receive and send chemical and mechanical signals, affecting thereby the functional adhesion complexes in many ways.

Integrin-related adhesion is an important event for many cells, including neurons and glial cells in the central nervous system (CNS). Our laboratory has previously reported that endogenous Thy-1 (CD90) expressed by neurons directly binds to the α_*V*_β_3_ integrin receptor in astrocytes, triggering downstream signaling events in these cells. The signaling pathways induce the formation of focal adhesions, increasing cell adhesion to the ECM (Leyton et al., 2001; Avalos et al., 2004; Hermosilla et al., 2008; Burgos-Bravo et al., 2020). These focal points are linked to stress fibers, which generate cell contractile forces through bundles of microfilaments associated with cytoskeletal proteins, such as myosin II (Stachowiak et al., 2012). The tensional forces generated within the cytoskeleton and applied to the ECM result from binding interactions between actin filaments and myosin. The phosphorylation of the regulatory myosin light chain (MLC) promotes these actin-myosin interactions, driving actin filament sliding (Polte et al., 2004). Likewise, the formation of focal adhesions and stress fibers depend on the phosphorylation of the MLC at Thr18/Ser19 (Chrzanowska-Wodnicka and Burridge, 1996).

On the other hand, primary astrocytes only respond to Thy-1 when exposed to a pro-inflammatory environment, such as that of a culture medium containing Tumor Necrosis Factor (TNF) (Lagos-Cabré et al., 2017). Therefore, Thy-1-integrin-induced astrocyte adhesion to the ECM is relevant in the context of pathological conditions. After a lesion or a disease affecting the CNS, several extracellular signals modulate the microenvironment. Glial cells in the brain contribute to these changes by producing and secreting inflammatory molecules and ECM proteins to modify ECM composition and stiffness (Hara et al., 2017). Therefore, astrocytes are subjected to chemical and physical signals in an inflammatory environment.

Astrocytes respond to injury and disease through a process known as astrocyte reactivity (Sofroniew and Vinters, 2010), which is not a simple all-or-none phenomenon. Instead, it is a finely graded series of changes that include reversible alterations in gene expression, cell hypertrophy and scar formation (Anderson et al., 2014). Hara et al. (2017) suggested the existence of a temporal sequence in the progression from naïve to reactive, and then from reactive to scar-forming astrocytes. They also indicated that astrocytes are motile and can revert to the non-reactive stage only in the reactive stage, but not once they progress to the scar-forming phase. Since both primary astrocytes treated with TNF and the DITNC1 astrocyte cell line migrate in response to Thy-1 (Kong et al., 2013; Lagos-Cabré et al., 2019), both cell types can be considered to be in the reactive stage.

Our recent reports propose that β_3_ Integrin is a key protein in the acquisition of the reactive phenotype in TNF-treated primary astrocyte cultures. The studies performed with astrocytes isolated from neonatal rat cortices show that TNF-induced cell reactivity leads to elevated expression of β_3_ integrin, Syndecan-4, GFAP, iNOS, Pannexin-1, and Connexin-43 (Lagos-Cabré et al., 2017), some of which are well-recognized markers of astrocyte reactivity. Therefore, considering that reactive astrocytes are responsible for the synthesis/secretion of several membrane proteins and ECM molecules, chemical and physical changes in the ECM after CNS injury might exert an effect on glial cells. However, the role of these mechanical signals in astrocyte reactivity has not been clearly defined yet. Hence, here we aimed to elucidate how reactive astrocytes can sense and respond to mechanical stress through integrins in an outside-in and inside-out manner.

Our study sheds light on how mechanical tension affects astrocyte responses to a chemical signal (Thy-1), which induces integrin ligation and thereby, leads to cell adhesion (increased focal adhesion and stress fiber formation) and contraction. These new/bigger focal adhesions translate into cytoskeletal changes and then into traction forces exerted by astrocytes onto the ECM. Here, our approach to study astrocyte mechanotransduction, complements classic cell biology methods with cutting-edge mechanobiology techniques. Considering that upon Thy-1–α_*V*_β_3_ Integrin-mediated cell-cell association, differentiated neurons retract their processes (Herrera-Molina et al., 2012; Maldonado et al., 2017), likely pulling on astrocyte integrins, we here applied external forces with integrin ligand-coated magnetic beads to mimic traction forces exerted by the neuron-bound chemical ligand (Thy-1) on astrocyte integrins. We also measured traction forces exerted by astrocytes onto the ECM through DNA-based tension gauge tethers (TGT). Therefore, the effect of neuronal Thy-1 on α_*V*_β_3_ Integrin would be promoted by traction forces from the neurons and the ECM to accelerate the formation of focal adhesions and astrocyte contraction. The findings aid in understanding the pathophysiological events occurring during insults to the CNS, the role these changes play in determining the rigidity of the ECM, and the forces generated by cell-cell communication. In a neuron-to-astrocyte context, forces generated by the ECM would exert an elevated tension on astrocytes, which will affect astrocyte communication with neighboring neurons. This mechanism represents another manner in which astrocytes in the glial scar prevent neuronal repair upon brain damage.

## Materials and Methods

### Cell Line

The rat astrocytic cell line DITNC1 (ATCC CRL-2005) was obtained from P. Magistretti (University of Lausanne, Switzerland), maintained in RPMI medium 1640 (GIBCO, Pittsburgh, PA, United States) containing 5% fetal bovine serum (FBS, HyClone, Pittsburgh, PA, United States), 0.1 mM 2-mercaptoethanol (GIBCO) and 100 U/ml penicillin/100 μg/ml streptomycin (PS mixture, GIBCO). DITNC1 astrocytes were maintained in culture at 37°C in a 5% CO_2_ humidified atmosphere. The medium was changed twice a week and passages were carried out by detaching the cells with 0.1% Trypsin (Invitrogen, Grand Island, NY, United States).

### Fusion Proteins and Magnetic Beads

Thy-1-Fc and TRAIL-R2-Fc fusion proteins were obtained as described previously (Leyton et al., 2001; Hermosilla et al., 2008; Avalos et al., 2009). These fusion proteins were incubated with magnetic beads coated with Protein A of 4.2 μm (Spherotech Inc., IL, United States) for 1 h at 4°C, prior to their use. Of note, TRAIL-R2-Fc is a fusion protein of the receptor for the soluble apoptosis-inducing ligand, TRAIL, and contains the Fc portion of the human IgG_1_. TRAIL-R2-Fc is used in these assays as a negative control.

### Pull-Up Traction Assay

DITNC1 cells (200.000 cells) were seeded on a 12 mm glass coverslip overnight for indirect immunofluorescence, or in 35 mm cell culture plates (Corning) for flow cytometry or Western blot experiments. Cells were serum deprived for 30 min prior to stimulation. Protein A-conjugated magnetic beads (diluted 1/4 in PBS) were incubated with Thy-1-Fc (5.8 μg of Thy-1 in a total volume of 20 μl) for 60 min on a rotating wheel at 4°C. The beads were allowed to bind to the surface of astrocytes for 5 min and then pulled with a magnetic field generated by a neodymium permanent magnet (N42 grade; K&J Magnets). After magnetic force application for different times (5, 10, 15, 30, and 45 min), the cells were prepared for analysis using the different assays.

### Cell Cytometry

Cells were detached using Trypsin/EDTA and incubated at 4°C to avoid internalization of surface proteins. After blocking with 5% Bovine Serum Albumin (BSA, Sigma-Aldrich, St. Louis, MO, United States), cells were immune-labeled with anti-α_*V*_β_3_ Integrin/Phycoerythrin (Santa Cruz Biotechnology, Dallas, TX, United States) for 2 h. Cells were analyzed using a FACS Canto (BD Bioscience, Franklin Lakes, NJ, United States) flow cytometer. Data were analyzed and plotted using FlowJo software (version vx).

### Western Blot Analysis

Protein extracts were prepared in lysis buffer (150 mM NaCl, 0.1% SDS, 0.25% sodium deoxycholate, 1% Triton-X100, in 50 mM Tris–HCl pH 7.4), supplemented with a protease and phosphatase inhibitor cocktail (Biotool, Houston, TX, United States). Extracts were electrophoretically separated on 10% or 12% SDS-PAGE gels and transferred to nitrocellulose membranes (Millipore, Billerica, MA, United States). The membranes were then blocked with 5% w/v BSA Fraction V in PBS containing 0.1% Tween-20, and subsequently incubated with anti-Phospho-Myosin Light Chain II (Ser19) (Cell Signaling Danvers, MA, United States), total Myosin Light Chain II (Cell Signaling Danvers, MA, United States), or anti-HSP90 (Santa Cruz Biotech) primary antibodies. The membranes were then washed and incubated with horseradish peroxidase-conjugated goat anti-rabbit IgG (Jackson ImmunoResearch Labs, Inc., West Grove, PA, United States) or goat anti-mouse IgG polyclonal antibody (Bio-Rad Laboratories, Inc., Hercules, CA, United States) for 1 h at room temperature. Bands were visualized with a chemiluminescence kit (Pierce, Thermo Scientific, Rockford, IL, United States), using an enhanced ECL detection system (GE Healthcare).

### Indirect Immunofluorescence

DITNC1 cells (20.000 cells) were seeded on 12 mm coverslips and left to adhere for 24 h. After different stimuli, cells were washed and fixed, as previously described (Avalos et al., 2009). After removing the fixative agent, cells were stained with anti-phosphotyrosine antibody (Abbkine, Australia), followed by secondary antibody conjugated to IF546 (Abbexa, Cambridge, United Kingdom). Astrocytes were stained with phalloidin/FITC (Molecular Probes, Eugene, OR, United States), and DAPI (diamidino- 2-phenylindole) (Sigma-Aldrich). Samples were visualized using a confocal Nikon Spectral C2 + Plus microscope (Nikon, Tokyo, Japan).

### Focal Adhesion Quantification

Quantification of the number and area of focal adhesions was performed using the ImageJ program with the “Analyze particle” tool, as reported previously (Sbalzarini and Koumoutsakos, 2005; Avalos et al., 2009).

### Stress Fiber Quantification and Analysis

F-actin fibers (number, coherency and average thickness) were examined in primary astrocytes using Hessian matrix-based analysis, as previously reported (Tanaka et al., 2019). The analysis was performed using the ImageJ “line” tool and the plugin FeatureJ. Actin thickness was calculated by measuring the integrated density fluorescence, corrected to fiber area. This measurement was adapted from Sathyanesan et al. (2012), who described nerve fiber quantification. Here, for actin fiber quantification, the histogram bin size was adapted to 2 arbitrary units of normalized fluorescence intensity.

### Tension Gauge Tether Probes

**FIGURE 1:**
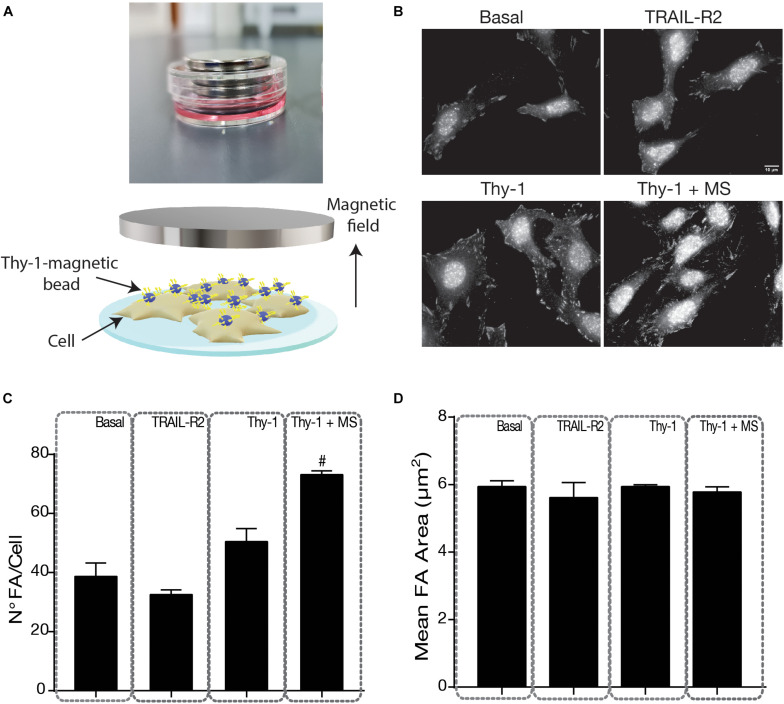
Indirect Immunofluorescence of focal adhesions in DITNC1 cells. **(A)** Image to the top shows the tissue culture plate with the magnets, which create the magnetic field. The scheme illustrates details of the experimental approach. Cells were plated on coverslips (light blue surface) and incubated with serum-free medium for 30 min. DITNC1 cells (beige) were stimulated with TRAIL-R2- (as a negative control) or Thy-1-coated Protein A-conjugated magnetic beads (blue spheres with yellow sticks), with or without mechanical stress (MS) applied by a magnet (gray) for 5 min. **(B)** Focal Adhesions (FA) were stained with an antibody against phospho-tyrosine, followed by a secondary antibody. Magnification bar = 10 μm. **(C,D)** Values in the graph are the mean + s.e.m. from at least 30 cells that were measured per condition in each experiment (*n* = 3) of the number (N°) of FA per cell **(C)** and the average area (μm^2^) of the FA **(D)**. Statistical significance was calculated using the Kruskal–Wallis non-parametric test with Dunn’s multiple comparison. #*p* < 0.05, compared with the Thy-1 condition.

**FIGURE 2:**
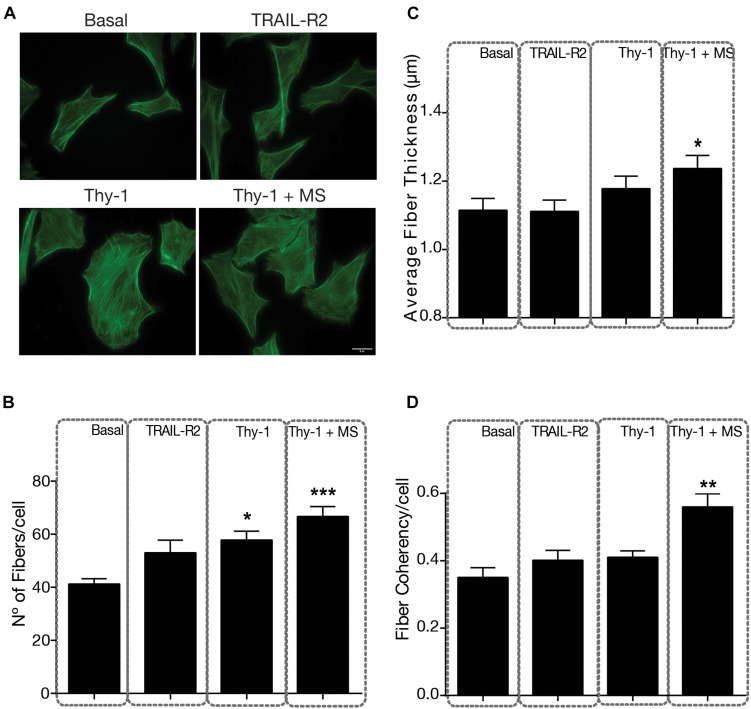
Indirect Immunofluorescence of stress fibers in DITNC1 cells. **(A)** Cells were plated on coverslips and incubated with serum-free medium for 30 min, and then stimulated as indicated in Figure 1, for 5 min. Stress fibers were labeled with phalloidin/FITC (green). Magnification bar = 8 μm. Values in the graph are **(B)** Number of stress fibers per cell, **(C)** Average thickness (μm) of stress fibers per cell, and **(D)** Stress fiber coherency (alignment) per cell. Values in histograms and error bars represent mean + s.e.m. from at least 30 cells that were measured per condition in each experiment (*n* = 3). Statistical significance was calculated using Kruskal–Wallis non-parametric test with Dunn’s multiple comparison. MS, Mechanical Stress. *p < 0.05, **p < 0.01, and ***p < 0.001, compared with basal conditions.

Tension gauge tether-modified DNA strands were synthesized and annealed according to previously established protocols (Supplementary Tables 1–3 and Figures 1, 2; Zhang et al., 2018). To prepare TGT-modified substrates, 75 mm × 25 mm glass coverslips were mounted on a teflon rack in a 40 ml beaker and rinsed three times in 40 ml of ethanol. Coverslips were sonicated in ethanol for 30 min, sonicated again in nanopure water (18.2 MΩcm^–1^) for 10 min and then dried in an oven at 100°C for 20 min. The cleansed slides were treated with fresh piranha solution (7:3 v/v = H_2_SO_4_: H_2_O_2_) for 10 min. CAUTION: piranha solution is highly oxidative and may violently explode if mixed with organic solvents such as ethanol or acetone. The coverslips were then rinsed six times with 40 ml of nanopure water, rinsed with ethanol to remove excess water and to further clean the substrates. Subsequently, 3% v/v (3-Aminopropyl) triethoxysilane (APTES) solution in ethanol was added to the slides, and incubated for 1 h. Afterward, the glass surface was rinsed with ethanol and dried in an oven set at 80°C for 30 min. Dried surfaces were incubated overnight with NHS-biotin (ThermoFisher Scientific 20217) (2 mg/ml in anhydrous DMSO) in a Petri dish at room temperature sealed with parafilm and used within 2 weeks of preparation. On the same day of TGT experiments, substrates were rinsed with ethanol and dried under a stream of N_2_ gas. Coverslips were then mounted in an Attofluor^TM^ cell chamber (ThermoFisher Scientific A7816) for microscopy and washed with PBS. The mounted surfaces were incubated with 0.1% BSA for 30 min, washed with 3 ml PBS and then covered with a 50 μg/ml solution of streptavidin in PBS. After incubating the coverslips for 1 h at room temperature, they were rinsed in 3 ml of PBS to remove the unbound streptavidin. The surfaces were then covered with 0.5 ml of 60 nM TGT DNA in PBS for 1 h. Unbound ligand was washed away with PBS and the surfaces were used within the same day of preparation.

### Statistical Analysis

Results were compared using the Kruskal–Wallis test and Dunn’s post-test with the GraphPad Prism 5 software (San Diego, CA, United States). The Mann-Whitney *U* test was employed for paired groups. The specific test used in the statistical analyses is indicated in the figure legends. Values averaged from three or more independent experiments were compared. *p* values < 0.05 were considered statistically significant.

## Results

### Astrocyte Adhesion and Contractility Induced by Thy-1 Stimulation Is Enhanced and Accelerated by Mechanical Stress

Our laboratory has described a role for Thy-1 in integrin ligation. Upon binding to integrins, Thy-1 increases integrin expression and clustering, focal adhesion and stress fiber formation, as well as cell contractility and migration of astrocytes (Avalos et al., 2004; Lagos-Cabré et al., 2017, 2018) and cancer cells (Brenet et al., 2020). Mechanical tension generated by a stiffer ECM also drives clustering of integrins (Cluzel et al., 2005). Therefore, it is possible that mechanical stress produced through structural ECM remodeling by proteins secreted from astrocytes or other cells after brain damage (Stichel et al., 1995; Haas et al., 1999; McKeon et al., 1999; Iseki et al., 2002; Liesi and Kauppila, 2002; Hagino et al., 2003; Hirano et al., 2004; Yiu and He, 2006; Gris et al., 2007; Falo et al., 2008; Bellin et al., 2009), could stimulate and prime the cells to respond to Thy-1.

#### Mechanical Stress Increases the Number of Focal Adhesions Induced by Thy-1

Focal adhesions are stabilized on stiff but not soft substrata, which thereby influence cytoskeletal structure and signaling (Moore et al., 2010; Chanet and Martin, 2014). Therefore, given the critical role of mechanical forces in focal adhesion formation (Bershadsky et al., 2003; Berrier and Yamada, 2007), we first tested how Thy-1-stimulated focal adhesions were affected by mechanical forces. Tension was generated by Thy-1-Protein A-coated magnetic beads, which were subjected to forces provoked by a magnet (Figure 1A). To this end, we evaluated the presence of these structural ECM contacts by indirect immunofluorescence (IIF). Focal adhesions are enriched in proteins phosphorylated on tyrosine (Chrzanowska-Wodnicka and Burridge, 1996); hence, we labeled them by staining with anti-phospho-tyrosine antibodies (Figure 1B). Basal levels of focal adhesions and those observed after treatment with TRAIL-R2-Fc averaged approximately 40 ± 6.7 FA/cell and no significant differences were found among these values (Figure 1C). TRAIL-R2-Fc is a fusion protein used as a control for the Fc portion of the Thy-1-Fc fusion protein (Avalos et al., 2009). TRAIL-R2-Fc bound to Protein A-conjugated magnetic beads, with or without a magnetic field, was used as a negative control in most of the experiments. The number of focal adhesions in astrocytes was unaffected when cells were stimulated with Thy-1-Protein A-magnetic beads for 5 min and no mechanical force was applied, but significantly increased (*p* = 0.0490) following the mechanical pulling of Thy-1-Protein A-magnetic beads (Figure 1C). Of note, the magnitude of force levels applied to pull the Thy-1 magnetic beads bound to the cell surface was the same as that reported by Fiore and coworkers (Fiore et al., 2015). In their study, they estimated a range between 10 and 16 pN of traction force per bead. Interestingly, while the number of focal adhesions increased with mechanical stress in this range, the size of these structures showed non-significant differences among conditions (Figure 1D). Noteworthy, our previously published results indicate that a significant effect of Thy-1 (coupled to either soluble Protein A or Protein A-conjugated to Sepharose beads) on focal adhesion formation is observed only after 10–15 min of stimulation (Henriquez et al., 2011; Kong et al., 2013). Therefore, at the time point tested here (5 min), the chemically induced signaling, which involves phospho-tyrosine, is enhanced only if a mechanical cue is also applied.

Mechanical stress increases the number, thickness, and alignment of stress fibers. Focal adhesions localize at the tip of stress fibers, accounting for its association with cell contractility and movement (Burridge and Guilluy, 2016). Therefore, we also tested stress fiber formation by staining these structures with FITC-conjugated phalloidin (Figure 2A). Stress fiber number (Figure 2B), thickness (robustness) (Figure 2C) and alignment (coherency) (Figure 2D) have been shown to be associated with cell tension and can be quantified using confocal microscopy, combined with image processing (Tanaka et al., 2019). The stimulation only with Thy-1 increased stress fiber number per cell (58 fibers/cell) compared to basal values (41 fibers/cell), but the increase was greater when Thy-1 was combined with a mechanical stimulus (67 fibers/cell) (Figure 2B); in both cases, values were significantly different to basal levels (*p* = 0.0103 and *p* = 0.0002, respectively). Moreover, stress fiber thickness and alignment were also significantly increased by the combination of Thy-1 and mechanical stress, compared to controls (Figures 2C,D). Interestingly, stimulation only with Thy-1 also increased these two stress fiber parameters, but not significantly (Figures 2C,D), suggesting that cell contractility is achieved more robustly when both Thy-1 and mechanical tension are applied together. Therefore, the behavior of stress fibers when stimulated with Thy-1 and force is similar to that observed for focal adhesions under the same conditions (compare Figures 1 and 2).

#### Phosphorylation of Myosin Light Chain After Thy-1 Stimulation and the Application of Mechanical Stress

**FIGURE 3:**
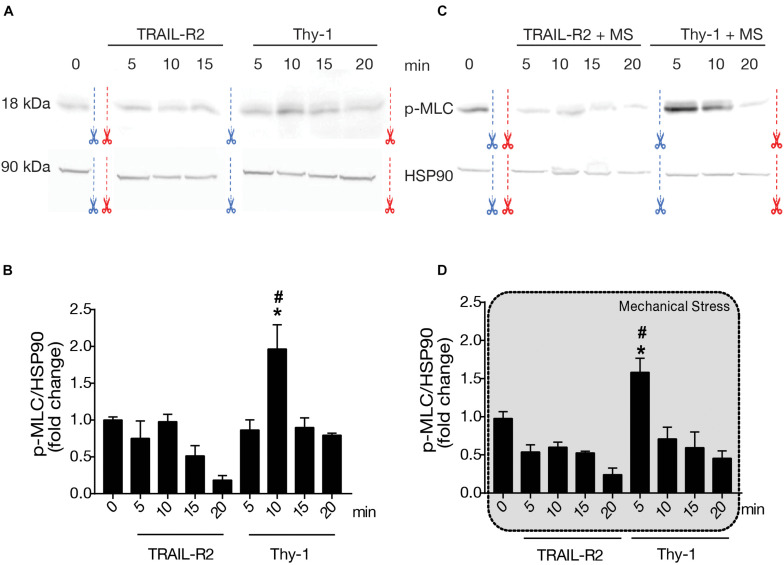
Phosphorylation of Myosin Light Chain after Thy-1 stimulation, with or without mechanical stress. DITNC1 cells were incubated with serum-free medium for 30 min and stimulated as indicated in Figure 1 for 5, 10, 15, and 20 min. **(A,C)** Whole cell lysates were separated by SDS-PAGE and transferred to nitrocellulose for immunoblotting. Bands of 18 kDa correspond to Myosin Light Chain phospho-Serine 19 (p-MLC) and those at 90 kDa, to the Heat Shock Protein 90 (HSP90). Blue and red scissors indicate where the figure (immunoblot image) was cut so that bands match the order of sample appearance in the corresponding graphs. **(B,D)** Values in the graphs correspond to densitometric analysis of the p-MLC band intensity normalized to Heat Shock Protein 90 (HSP90) values. The application of mechanical forces exerted by a magnet are indicated using a gray shaded background in graph D. Statistical significance was calculated using Mann-Whitney non-parametric test. * and #, *p* < 0,05; *compared with the basal condition at T = 0, # compared with the value obtained at the same time with TRAIL-R2.

The role of mechanical stress in astrocyte contractility induced by Thy-1 has not been studied. Therefore, to evaluate if cellular contractility increases at a molecular level, we measured the phosphorylation of MLC by immunoblotting. After applying a magnetic force for different time intervals (5, 10, 15, and 20 min), we found that stimulation with Thy-1 increased the levels of MLC phosphorylation on serine 19, compared to basal levels found under non-stimulated conditions or treatment with the negative control TRAIL-R2 (Figures 3A,B). In addition, mechanical stress accelerated MLC phosphorylation, since the peak of phosphorylation was observed earlier on after 5 min, rather than 10 min without mechanical stimulation (Figures 3C,D). With Thy-1 alone, MLC phosphorylation levels significantly increased at 10 min (*p* = 0.0143) compared to time 0 (Figure 3B); whereas, for Thy-1 + mechanical stimulation, the significant increase was found at 5 min (*p* = 0.0286) compared to time 0 (Figure 3D). This molecular data correlates well with the results presented in Figure 2, showing an effect on Thy-1-induced contractility only when the cells were stimulated with the combination of Thy-1 and mechanical stress for 5 min. Importantly, MLC phosphorylation was either not affected or even showed a tendency to reduced levels when magnetic beads were coated with the control protein TRAIL-R2-Fc, indicating that neither the fusion protein-Fc nor the magnetic field *per se* triggers the detected effect.

### Astrocytes Can Sense and Exert Mechanical Stress Through Integrins in an Outside-In and Inside-Out Manner

**FIGURE 4:**
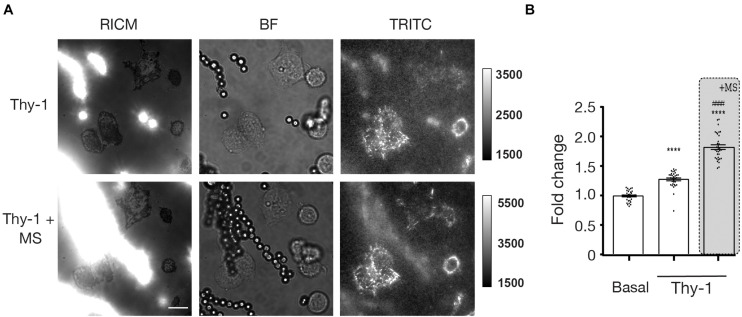
Traction forces are increased after Thy-1 and mechanical stress stimulation in astrocytes. **(A)** Representative images of DITNC1 cells stimulated only with Protein A-magnetic beads coated with Thy-1 (top row) or with Thy-1-Protein A-magnetic beads plus mechanical stress (MS) (bottom row). The left column corresponds to Reflection Interference Contrast Microscopy (RICM), the middle column shows Bright Field (BF), and the right column, Total Reflection Internal Fluorescence of Traction Forces (TRITC). Magnification bar = 12 μm **(B)** Quantification of mean fluorescence intensity measuring traction force for the basal, Thy-1 alone (for 5 min) and Thy-1 + mechanical stress (for 5 min) conditions (at least 30 cells per condition were counted). Values were normalized to the fluorescence intensity observed for cells without bead contact at the same time point for each condition (value + s.e.m. of three independent experiments). Statistical significance was calculated using the Kruskal–Wallis non-parametric test, with Dunn’s multiple comparison. ****p < 0.0001, compared with basal tension; ####*p* < 0.0001, compared with the tension corresponding to Thy-1 only.

Because Thy-1 induces cell adhesion and contractility by ligating α_*V*_β_3_ Integrin (Leyton et al., 2001), the results in this study suggested that mechanical stress applied through α_*V*_β_3_ Integrin triggered an outside-in response in astrocytes, which led to increased cellular adhesion and contraction. Considering the established ability of integrins to signal in an outside-in as well as inside-out manner (Mohammed et al., 2019), we then tested whether the rearrangement of the cytoskeleton could generate traction forces *via* focal adhesion integrins from within the cell and to the ECM outside the cell. To test this idea, we used recently developed DNA-based molecular tension sensors (Stabley et al., 2011; Zhang et al., 2014; Ma and Salaita, 2019) to quantify the forces exerted by astrocyte integrins onto the ECM ligands. As a first approach, we used the DNA hairpin probes (Zhang et al., 2014), which measure the molecular forces exerted by integrins in real time and with piconewton resolution (Supplementary Figure 3). These probes are comprised of a stem-loop DNA structure labeled with a fluorophore-quencher pair. In addition, the probes unfold reversibly in response to external forces that exceed the threshold value of 4.7 pN (Zhang et al., 2014; Liu et al., 2016). We used these probes to measure the forces exerted by DITNC1 cells stimulated with Protein A-magnetic beads coated with Thy-1 pulled with a magnet while in contact with the cell. The tension signal obtained in these samples was weak, suggesting that the density of mechanical events that led to hairpin unfolding was low. In addition, a negative signal appeared at potential points of integrin adhesions, which suggests degradation of the probe (Supplementary Figure 3, arrows). Thus, we next tested the tension gauge tether (TGT) probes which irreversibly generate a tension signal and hence, report on the accumulated history of mechanical events over time. This provides a mechanism to enhance the measured signal over time. The TGT is comprised of an immobilized DNA duplex that is engineered to denature at specific tension tolerance (*T*_*tol*_) magnitudes that range from 12 pN to 56 pN, depending on the pulling geometry (unzipping or shearing mode, respectively). Thermal melting of the TGT is identical in both cases and the threshold of *T*_*tol*_ is simply tuned by controlling force pulling orientation relative to the surface anchoring moiety. In the shearing geometry, mechanical melting of the duplex requires a *T*_*tol*_ = 56 pN, in contrast to the unzipping geometry, which requires a *T*_*tol*_ = 12 pN. To detect mechanically induced denaturation of the TGT duplex, we employed an oligonucleotide duplex with the stop strand presenting a cyclic arginine-glycine-aspartic acid-D-phenylalanine-lysine (cRGDfK) with a quencher (BHQ2) hybridized to a biotinylated strand. The biotin group anchors the oligonucleotide duplex to the substrate and presents a fluorophore (Cy3B) (Supplementary Figure 1), which is un-quenched upon tension-induced denaturation. In this experiment, we plated DITNC1 cells over both TGT probes, with a *T*_*tol*_ = 12 and 56 pN. Astrocytes displayed enhanced adhesion (visualized by spreading area and cell morphology) on the 56 pN TGT probe compared to the 12 pN TGT surface (Supplementary Figure 4) likely due to enhanced ligand mechanical strength as reported by previous studies (Wang and Ha, 2013). Thus, we selected the 56 pN probe for the subsequent experiments. We then measured the traction forces exerted by astrocytes by quantifying the fluorescence intensity under each cell. This intensity indicates the number of mechanical events that exceed the *T*_*tol*_ of 56 pN transmitted through integrins after Thy-1 mediated mechanical stimulation. Astrocytes were plated onto the 56 pN TGT probes for 30 min, stimulated with Thy-1-coated Protein A-magnetic beads for 5 min, and then stressed by applying a magnetic field for another 5 min. Magnetic beads and the cell adhesion regions were visualized using Reflection Interference Contrast Microscopy (RICM), where the magnetic beads appeared as bright spots due to light scattering (Figure 4A, left panels). Brightfield (BF) was used to visualize the magnetic particles as well as the cells. For the analysis, we only selected the cells that were in contact with the beads and values were normalized to tension levels determined for cells without beads at the same time point (Figure 4A, middle panels). The Cy3B fluorescence channel corresponded to forces exerted on cells through integrins (Figure 4A, right panels). The normalized Cy3B data showed that Thy-1 stimulation at *t* = 5 min significantly increased integrin forces applied by cells, by 1.28-fold compared to control cells (*p* < 0.0001) (Figure 4B). Importantly, when mechanical stimulation through Thy-1 was applied for the same period of time, the TGT signal was significantly enhanced by ∼1.9-fold (*p* < 0.0001) (Figure 4B). These results indicate that integrins transduce chemical and mechanical signals not only in an outside-in, but also in an inside-out manner, by exerting forces from the cytoskeleton back to the ECM.

### Thy-1 Plus Mechanical Stress Increase Surface Levels of α_*V*_β_3_ Integrin Faster Than Thy-1 Alone

**FIGURE 5:**
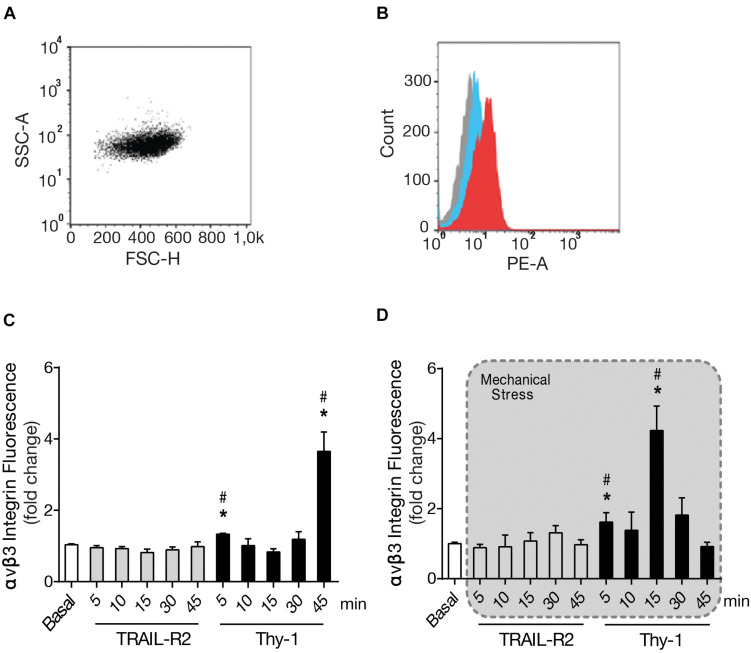
Thy-1 plus mechanical stress increase surface levels of α_*V*_β_3_ Integrin faster than Thy-1 alone. **(A)** Forward and side scattering (FSC-H and SSC-A) gating shows the selected cell population, based on size and granularity. **(B)** The three cell populations shown correspond to non-labeled control (gray), isotype control (cyan) and α_*V*_β_3_ Integrin/Phycoerythrin (red). **(C,D)** DITNC cells were incubated with serum-free medium for 30 min and stimulated with Protein A-magnetic beads coated with Thy-1, with and without mechanical forces exerted with a magnet for 5, 10, 15, 30, and 45 min. Values in the graph are the mean fluorescence intensities normalized to the control signal (basal), TRAIL-R2 is a negative control in experiments with Thy-1. Values in the graph are the mean fluorescence intensities of α_*V*_β_3_ Integrin/Phycoerythrin, normalized to basal levels without stimuli. Values in the graphs represent mean + s.e.m. (*n* = 3). Statistical significance was calculated using Kruskal–Wallis non-parametric test, with Dunn’s multiple comparison. * and #, *p* < 0,05. * compared to the basal condition, # compared to TRAIL-R2 incubated for the same time.

Considering that the ECM is altered upon CNS damage, increasing the mechanical forces applied on cells, and that integrins are receptors for ECM proteins, as well as mechanoreceptors, we set out to study the effect of mechanical stress on integrin expression levels. Particularly because, we have previously reported increased cell surface α_*V*_β_3_ Integrin levels in astrocytes as a consequence of TNF-induced reactivity, and even further, by Thy-1 stimulation (Lagos-Cabré et al., 2018). Therefore, we studied possible changes in integrin levels when applying mechanical stressors. To this end, we used Protein A-magnetic beads coated with Thy-1-Fc. The beads were left to bind to the astrocyte surface and then pulled with a magnet. We used cell cytometry to measure the surface levels of α_*V*_β_3_ Integrin and first selected the cell population by volume and granularity (Figure 5A). This selection was maintained for all samples. The different probes used to identify α_*V*_β_3_ Integrin and the isotype control were measured as the mean of fluorescence intensity (Figure 5B). We then tested the response of cells to stimulation with Thy-1 (Figure 5C), or Thy-1 plus mechanical stress (Figure 5D) after different times of magnetic force application (5, 10, 15, 30, and 45 min). Upon mechanical stimulation, we found surface levels of α_*V*_β_3_ Integrin that peaked with mechanical stimulation at shorter time points than with Thy-1 alone. With Thy-1 alone, integrin levels significantly increased at 5 (*p* = 0.0143) and 45 min (*p* = 0.0286) compared to the basal condition (Figure 5C). Alternatively, with Thy-1 + mechanical stimulation significant increases were observed after 5 (*p* = 0.0283) and 15 min (*p* = 0.0286) compared to basal levels (Figure 5D). When the cells were treated with the negative control, the surface levels of α_*V*_β_3_ Integrin remained similar to basal levels. These results indicate that mechanical stimulation of Thy-1-engaged receptors enriches α_*V*_β_3_ Integrin astrocyte surface presentation compared to cells stimulated with Thy-1 only.

## Discussion

In this study, we explored a novel response of astrocytes to physical sensing of their environment, and particularly, the role of the mechanoreceptor α_*V*_β_3_ Integrin. Here, we verified that Thy-1, as a ligand for α_*V*_β_3_ Integrin receptor, is an important promoter of astrocyte contraction and introduced the novel concept that the addition of mechanical stress accelerates the formation of contractile structures, such as focal adhesions and stress fibers, in an outside-in manner. The appearance of α_*V*_β_3_ Integrin at the cell surface also occurs faster under stimulation with Thy-1 plus mechanical tension, likely facilitating integrin clustering and activation. As a consequence, astrocytes contract the cytoskeleton, exerting traction forces on the ECM through integrin in focal adhesions in an inside-out manner.

Clustering of integrins is a phenomenon that requires the lateral assembly of integrins in the plane of the plasma membrane, which occurs prior to focal adhesion formation; however, integrin activation is a prerequisite for integrin clustering (Cluzel et al., 2005). Considering that mechanical stress caused by a stiffer ECM drives clustering of integrins (Paszek et al., 2009), it is possible that the tension generated by structural ECM remodeling after brain damage (Stichel et al., 1995; Haas et al., 1999; McKeon et al., 1999; Iseki et al., 2002; Liesi and Kauppila, 2002; Hagino et al., 2003; Hirano et al., 2004; Yiu and He, 2006; Gris et al., 2007; Falo et al., 2008; Bellin et al., 2009) contributes to preparing the cells to respond to Thy-1 in a manner that synergizes with the effect of inflammation (TNF). The latter is supported by the fact that cells, which do not contain a threshold level of surface integrins, do not respond to Thy-1 (Lagos-Cabré et al., 2017). The effect of mechanical stress on astrocytes could be additional or parallel to the effect of pro-inflammatory cytokines, which are also released upon brain damage. Thus, in astrocytes, integrin clustering would be promoted by Thy-1 in an inflammatory environment and facilitated by mechanical stress.

We showed that surface levels of α_*V*_β_3_ Integrin, cellular contraction, and phosphorylation of MLC were elevated after combining Thy-1 stimulation with mechanical stress. These responses were faster than those induced by stimulation with only Thy-1. Perhaps the force application prolongs the bond lifetime of the interacting molecules, a phenomenon known as “catch-bond” behavior; however, our reported data indicate that the Thy-1-α_*V*_β_3_ Integrin interaction shows a bond lifetime that behaves as a “slip-bond”; namely, an applied force accelerates the dissociation between these two molecules (Burgos-Bravo et al., 2018). Because these results on bond properties were obtained using purified proteins in a single molecule scheme, it is possible that other molecules are involved in a cell-cell interaction. Indeed, we have reported that not only integrin, but also Syndecan-4 is required for Thy-1-induced astrocyte adhesion and migration (Avalos et al., 2009). Furthermore, using a different cellular model, Barker et al. reported that the Thy-1-α_5_β_1_ Integrin interaction exhibits a slip-bond behavior, which converts to a catch-bond behavior when Syndecan-4 is introduced into the cellular system (Fiore et al., 2014). Thus, it is possible that force applied to a tri-molecular complex—rather than to a bimolecular interaction—accounts for a faster response when a mechanical force is applied. However, our recently published data indicate that the slip-bond Thy-1-α_*V*_β_3_ Integrin interaction remains as a slip-bond when Syndecan-4 is introduced into the Optical miniTweezers system (Burgos-Bravo et al., 2020). In addition, under *in vitro*-generated pro-inflammatory conditions, Syndecan-4 accelerates the effect of integrin-engaged Thy-1 by forming a ternary complex, leading to a faster neuronal response, a phenomenon also related to cell contraction (Burgos-Bravo et al., 2020). Similar contractile outcomes could also occur in astrocytes upon the formation of the trimolecular complex; however, this aspect requires further investigation.

**FIGURE 6:**
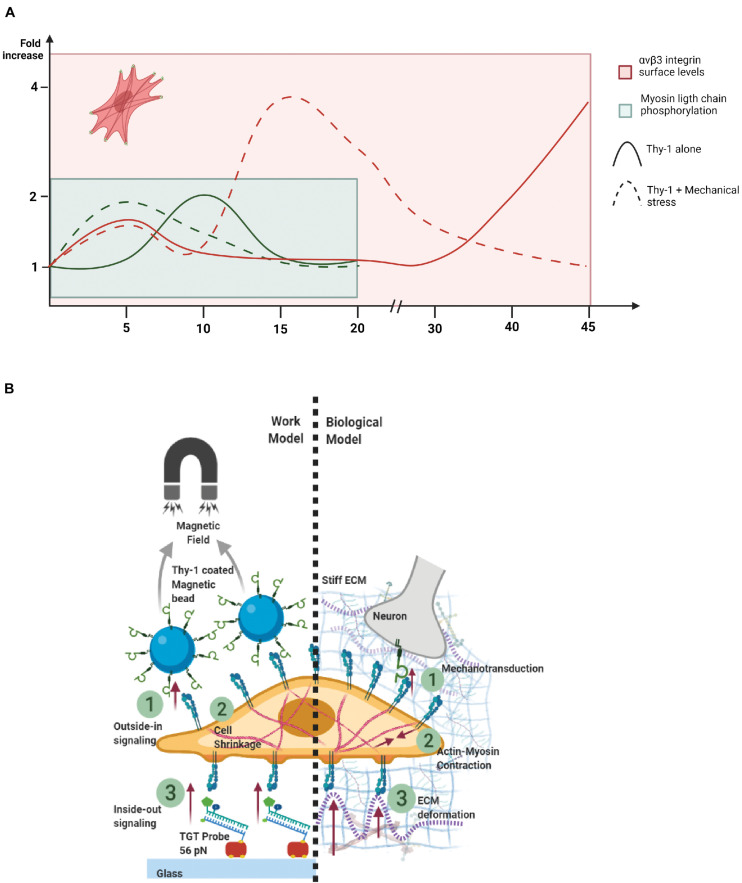
Scheme of outside-in switch in integrin signaling caused by mechanical stress. **(A)** Summary of the kinetics of astrocyte molecular/cellular responses. Dashed lines show the curve of Thy-1 plus mechanical stress stimulation, solid lines show Thy-1 alone stimulation over time. Red lines represent the kinetics of α_*V*_β_3_ Integrin surface levels, while green lines show the kinetics followed by the Myosin Light Chain phosphorylation. Red cell, with darker red lines and green spots, represents a cell with an increased number of focal adhesions and stress fibers at 5 min of stimulation compared with the non-stimulated control. **(B)** Work Model: (1) Outside-in signaling through α_*V*_β_3_ Integrin stimulated chemically by Thy-1 and mechanically by a magnetic field. (2) Cellular shrinkage as a response to integrin activation. (3) Inside-out signaling through integrins, where the astrocyte exerts tension force to the TGT probe-coated glass. Biological Model: (1) Mechanotransduction through the α_*V*_β_3_ Integrin, triggered by a stiff ECM (cell-matrix) and neuronal Thy-1 (cell-cell) interaction, leading to (2) Actin-myosin contraction and traction force exerted by the astrocyte through Integrins generate (3) ECM deformation. Both figures were created with BioRender.com.

Our previous reports showing that integrin surface availability is increased after pro-inflammatory stimuli (TNF) in primary astrocytes also showed a crowding phenomenon of integrins after TNF treatment and Thy-1 stimulation (Lagos-Cabré et al., 2018). Because of this possibility, mechanical stimulation could also increase integrin aggregation through the crowding caused by Thy-1-coated Protein A-magnetic beads. In this context, we have reported that higher RhoA activation is achieved when using Thy-1 conjugated to Protein A-Sepharose beads than when using soluble Thy-1 because higher integrin clustering is attained when presenting Thy-1 in a multivalent fashion (Avalos et al., 2004). The RhoA GTPase is an upstream activator of Rho-associated kinase (ROCK), which phosphorylates myosin phosphatase-targeting subunit 1 (MYPT1), thus decreasing MLC phosphatase activity and increasing MLC phosphorylation. Additionally, ROCK also phosphorylates MLC, leading to increased cellular contraction (Yamaguchi et al., 2006). Therefore, since RhoA activation is upstream of MLC phosphorylation (Avalos et al., 2004), the possibility that mechanical stimulation induces integrin crowding and faster RhoA activation is also feasible. If we recapitulate the events triggered by both Thy-1 stimulation and mechanical stress and compare them with Thy-1 stimulation only over time, integrin levels and MLC phosphorylation increase faster when the two stimuli are applied together (Figure 6A). Although cellular responses occur within a short time frame, the peak for the cellular contraction is faster than the main peak observed for the rise in integrin surface levels. However, a small increase in integrin levels is also seen after 5 min (Figure 6A), which could explain the initial contraction and faster focal adhesion formation. Additionally, once the contraction reaches a certain threshold, focal adhesions may continue oscillating for extended periods of time. The dynamic nature of focal adhesions allows them to act as mechanosensors of changes in ECM stiffness and do so in a highly flexible manner. This behavior facilitates outside-in and inside-out signaling through focal adhesion traction oscillations, in a cyclic manner (Wu et al., 2017). Therefore, focal adhesion dynamics, traction force-induced contractility and ECM stiffness act as sensors of the structural changes occurring in the cell microenvironment, all of which are considered indispensable for cell migration induced by changes in ECM stiffness (Plotnikov et al., 2012). Another possible explanation is that integrin activation due to Thy-1 binding is enhanced by mechanical stress. Because α_*V*_β_3_ Integrin is a mechanoreceptor, the mechanical stimulus would induce more α_*V*_β_3_ Integrin molecules to become activated, allowing them to maintain an active conformation (extended and open) faster than without mechanical stress (Chen et al., 2017). However, details about this process and the actual mechanism that explains it, remain elusive.

Astrocytes interact with neurons, blood vessels, and ECM proteins, and respond and adapt to the physicochemical modifications generated by an inflammatory environment. The adaptive response involves many changes in gene expression (Iacobas et al., 2020), and integrins are one of the key surface proteins which are upregulated in inflammation-mediated astrocyte reactivity (Lagos-Cabré et al., 2017). Importantly, integrins are the cell-to-matrix interface, which plays an important role in outside-in and inside-out signaling (Jaalouk and Lammerding, 2009). In our model system of neuron-astrocyte interaction, integrins play a key role in cell-cell interaction as well, and neuronal process retraction due to Thy-1-α_*V*_β_3_ Integrin binding (Maldonado et al., 2017) would cause integrin pulling forces. Force-induced mechanotransduction in an outside-in manner (cell-to-cell) changes cellular contractility, spreading, and adhesion, triggering cellular responses, thereby leading to inside-out signaling (cell-to-matrix), changing the topography, rigidity, and confinement of a cell. However, these responses in astrocytes have thus far, not been investigated. Here, we investigated the response of traction forces exerted by astrocytes through focal adhesion integrins onto the substrate (ECM). From our previous reports, we know that focal adhesions formed by Thy-1-ligated integrins occur by engaging α_*V*_β_3_ Integrin (Leyton et al., 2001; Hermosilla et al., 2008). However, focal adhesions that transfer contractile forces from the cell to the ECM could involve α_*V*_β_3_/α_5_β_1_ Integrins, which are the two integrins present in astrocytes (Leyton et al., 2001) that form focal adhesions and bind to the cyclic RGD probe (Kapp et al., 2017) used in this study.

The data in the present paper reinforce the idea of cellular tensegrity proposed by Ingber (2003), in which case, cells not only sense the mechanical cues from their microenvironment, but also generate mechanical cues that are transmitted to the ECM that could remodel the surrounding area of the cells (Figure 6B). We show that the magnitude of traction forces exerted by astrocytes on the ECM depends on the mechanical and chemical stimuli received by the cells. Specifically, the stimulus from neurons (Thy-1) in the context of cell-to-cell communication and those produced by a proinflammatory environment after injury, which generate a stiffer ECM (Figure 6B, #1). Mechanical and chemical signals lead to actin-myosin contraction (Figure 6B, #2), and the traction force of astrocytes to the ECM through integrins leads to ECM deformation (Figure 6B, #3). Thus, the mechanotransduction signaling network in reactive astrocytes works through integrins, using an outside-in switch caused by chemical and mechanical forces. Additionally, these results reveal the importance of physical stressors from the ECM in the promotion of astrogliosis. Therefore, a stiffer matrix promotes astrocyte reactivity, and lessen the likelihood of achieving neuronal regeneration after injury.

## Data Availability Statement

The original contributions presented in the study are included in the article/Supplementary Material, further inquiries can be directed to the corresponding author/s.

## Author Contributions

LP and LL designed and executed the experiments, interpreted the data, and prepared the article. LP, ARa, JC, and ARo helped to execute the experiments and interpret the data. LP and ARa additionally prepared the data for publication and helped to write the article. LP, KS, PS, and LL worked on interpreting the data and preparing and writing the article. All authors have read and agreed to the submitted version of the manuscript.

## Conflict of Interest

The authors declare that the research was conducted in the absence of any commercial or financial relationships that could be construed as a potential conflict of interest.

## Publisher’s Note

All claims expressed in this article are solely those of the authors and do not necessarily represent those of their affiliated organizations, or those of the publisher, the editors and the reviewers. Any product that may be evaluated in this article, or claim that may be made by its manufacturer, is not guaranteed or endorsed by the publisher.
