# Supplementary material for: An Outside-In Switch in Integrin Signaling Caused by Chemical and Mechanical Signals in Reactive Astrocytes
Source: Front Cell Dev Biol. 2021 Aug 23;9:712627. doi: 10.3389/fcell.2021.712627 (PMC8419233; doi:10.3389/fcell.2021.712627)
Supplement: Supplementary file 1 [file Data_Sheet_1.pdf]

Supplementary Material

1. Supplementary Figures and Tables

Supplemental Note 1. TGT-modified DNA constructs

To create TGT-modified DNA constructs, modified DNA was purchased from Integrated DNA Technologies (IDT), and synthetically modified with Cy3B fluorophore and cRGD ligand (Table 1), as previously described [1], resulting in the 56 pN TGT construct shown in Figure 1 below.

Supplementary Table 1. Sequences of modified DNA purchased from Integrated DNA Technologies with modified bases enclosed in slashes.

**Strand Name**	**Sequence**
Top Strand	5'-/5Hexynyl/GTG AAA TAG CGC ACA GAT GCG /3-BHQ2-3'
Bottom Strand	5'-/5Biosg/TT T/iUniAmM/C GCA TCT GTG CGC TAT TTC AC-3'

Supplementary Figure 1

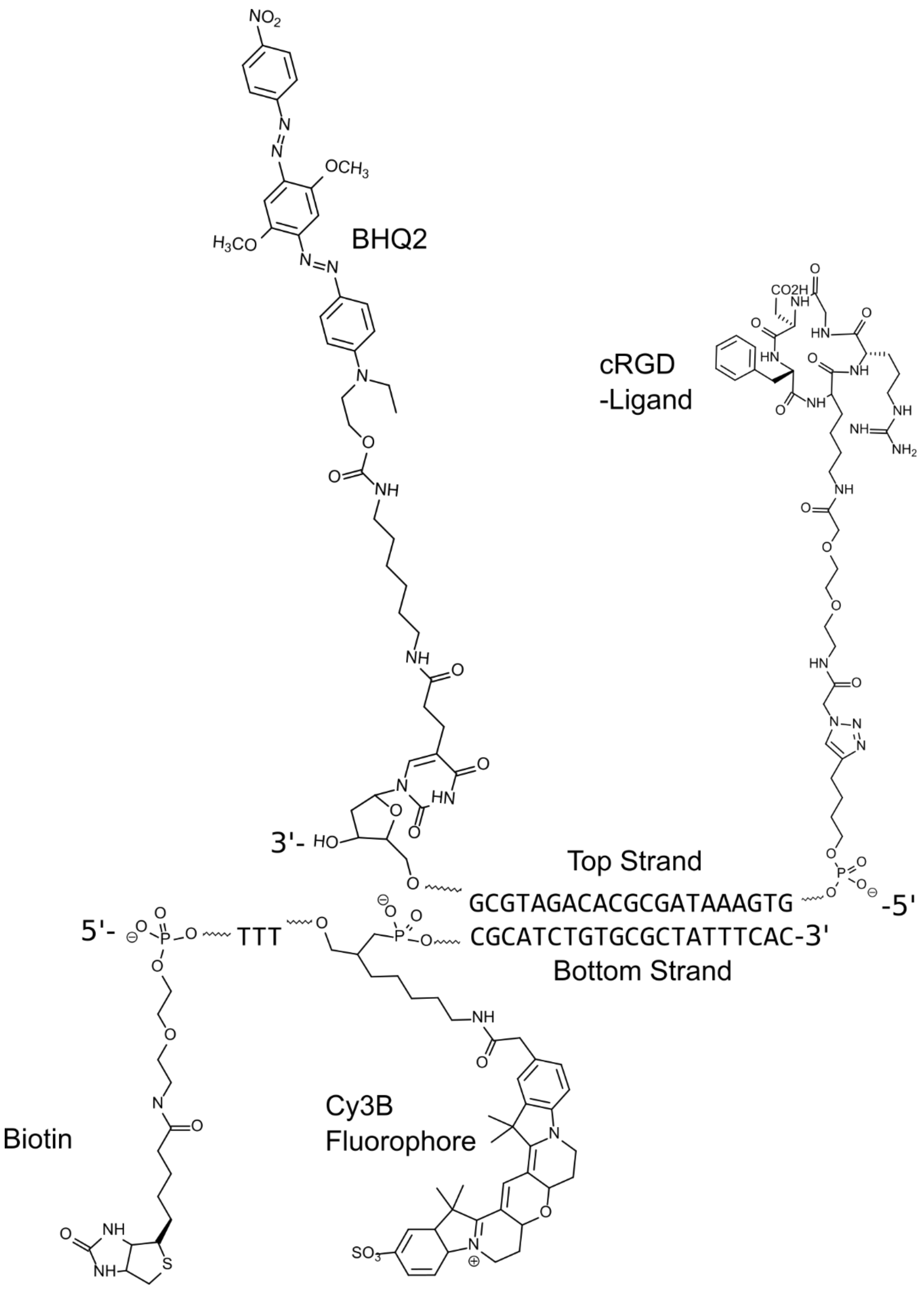

Supplementary Figure 1: Chemical structure and DNA sequence of the assembled 56 pN TGT construct.

Supplemental Note 2. Hairpin Probe Constructs

To create Hairpin-modified DNA constructs, modified DNA was purchased from Integrated DNA Technologies (IDT). Oligonucleotide sequences are summarized in tables 2 and 3. The hairpin construct of 4,7 pN is shown in figure 2 below.

Supplementary Table 2. Sequences for 4.7 pN hairpin probe before modification from IDT

**Strand Name**	**Sequence**
Ligand Strand	5’-/5Hexynyl/-TTT GCT GGG CTA CGT GGC GCT CTT–/3AmMO/-3’
4.7 pN Hairpin	5'-GTG AAA TAC CGC ACA GAT GCG TTT GTA TAA ATG TTT TTT TCA TTT ATA CTTT AAG AGC GCC ACG TAG CCC AGC -3’
Anchor BHQ	5’- / BHQ 1/-CGC ATC TGT GCG GTA TTT CAC TTT-/ Biotin/-3’

Supplementary Table 3. Sequences for 4.7 pN hairpin probe after modification

**Strand Name**	**Sequence**
Ligand Strand	5’-/cRGD/-TTT GCT GGG CTA CGT GGC GCT CTT–/Cy3B/-3’
4.7 pN Hairpin	5'-GTG AAA TAC CGC ACA GAT GCG TTT GTA TAA ATG TTT TTT TCA TTT ATA CTTT AAG AGC GCC ACG TAG CCC AGC -3’
Anchor BHQ	5’- / BHQ 1/-CGC ATC TGT GCG GTA TTT CAC TTT-/ Biotin/-3’

Supplementary Figure 2

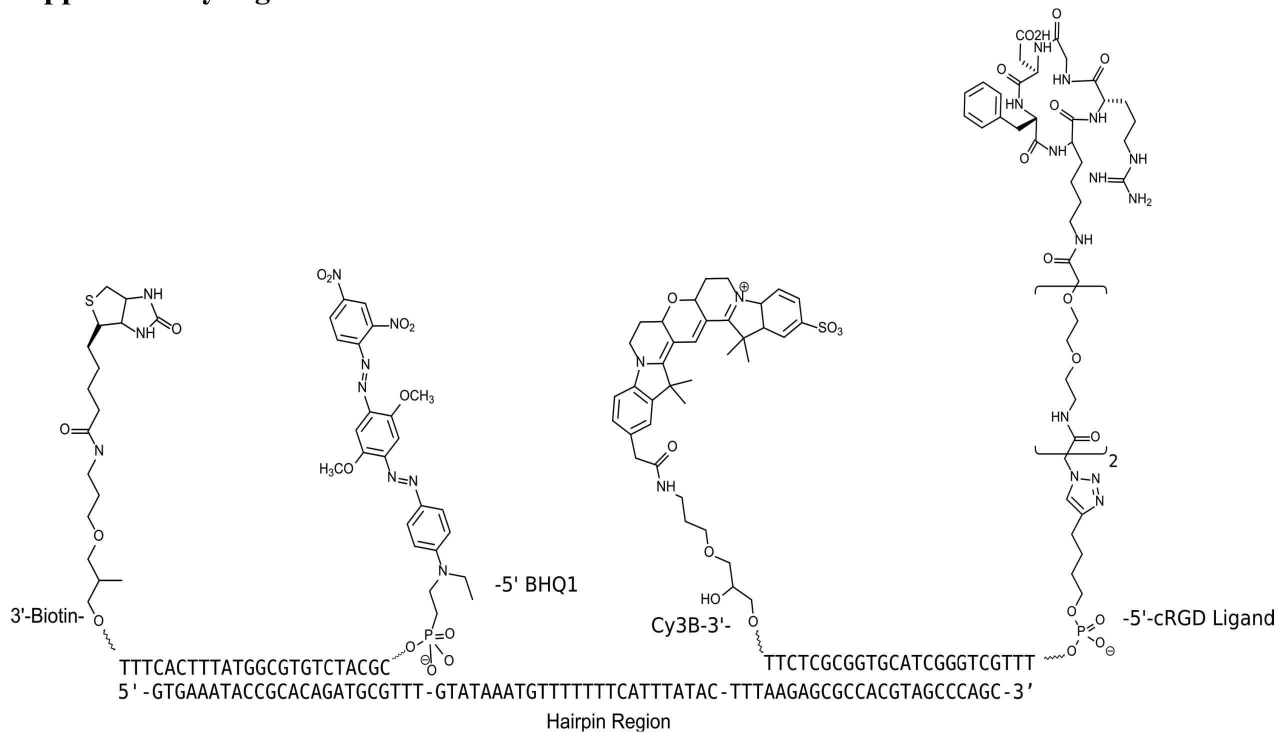

Supplementary Figure 2. Chemical structure and DNA sequence of the assembled 4.7 pN Hairpin Probe

Supplementary Figure 3

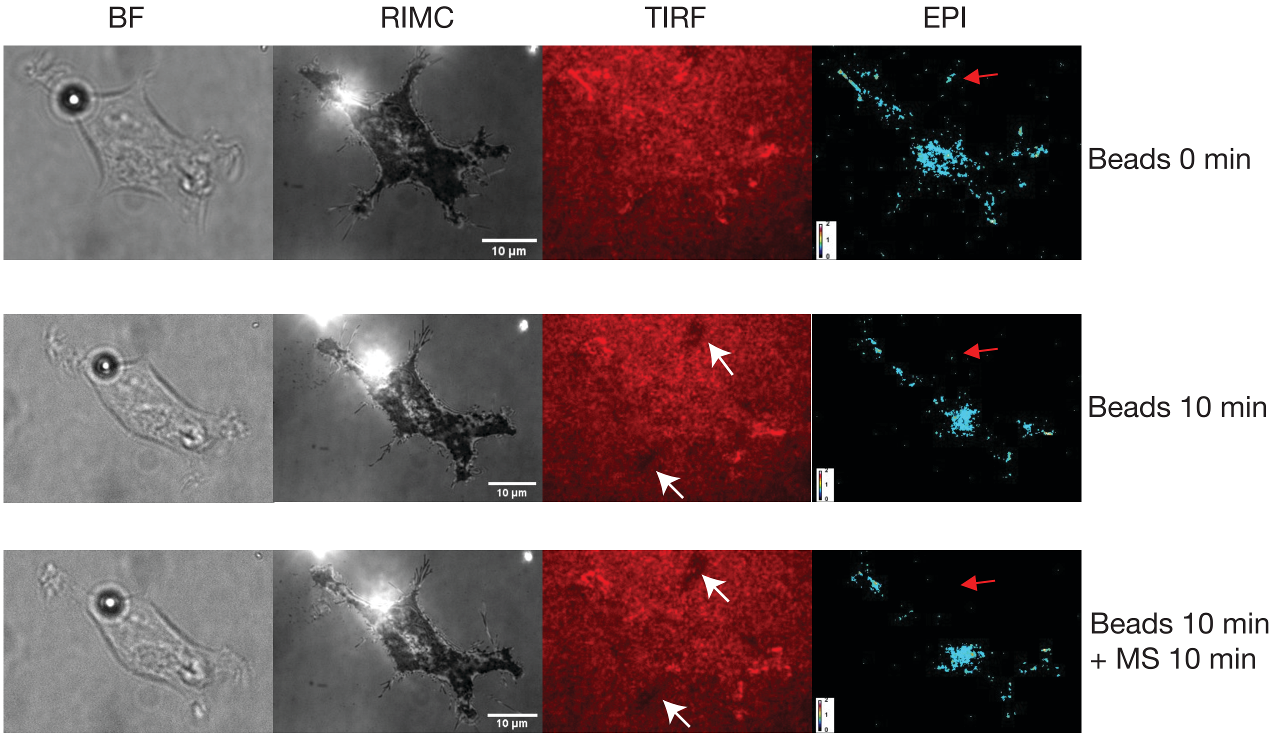

Supplementary Figure 3. Traction force microscopy of DITNC1 cells using a 4.7 pN hairpin probe. *Representative images of DITNC1 cells added over hairpin probes and stimulated with Thy-1-coated Protein A-magnetic beads and mechanical stress. Pictures obtained with Bright Field (BF), Reflection Interference Contrast Microscopy (RICM), Total Internal Reflection Fluorescence (TIRF) and Epifluorescence (EPI) channels are shown. The EPI channel shows the quantification of the % of open probes, as previously described [2]. Each condition shows dark spots in TIRF channels and a loss of tension signal in the EPI channel, which could correspond to DNA degradation by nucleases or disengagement of the probe (white and red arrows, respectively). Magnification bars=10 µm.*

Supplementary Figure 4

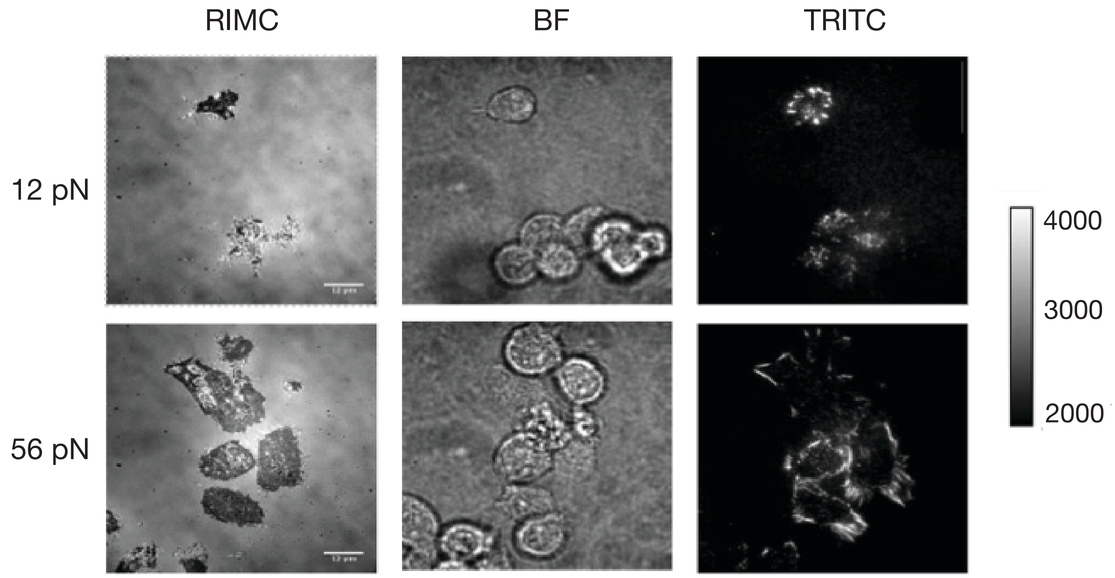

Supplementary Figure 4. Traction force microscopy of DITNC1 cells with 12 pN and 56 pN TGT probes. *Representative images of DITNC1 cells added over TGT probes. Microphotographs of Bright Field (BF), Reflection Interference Contrast Microscopy (RICM) and Total Internal Reflection Fluorescence (TRITC) channels are shown. Intensity fluorescence scale for TRITC is indicated at the right of the figure. The 56 pN TGT probe (bottom, row) shows greater cell spreading, a less rounded shape and an increased tension signal, compared to the 12 pN TGT probe (upper row). Magnification bar=12 µm*

References

1. Zhang, Y., et al., *Platelet integrins exhibit anisotropic mechanosensing and harness piconewton forces to mediate platelet aggregation.* Proc Natl Acad Sci U S A, 2018. **115**(2): p. 325-330.
2. Glazier, R., et al., *DNA mechanotechnology reveals that integrin receptors apply pN forces in podosomes on fluid substrates.* Nat Commun, 2019. **10**(1): p. 4507.
