# Supplementary figures and images for: An Outside-In Switch in Integrin Signaling Caused by Chemical and Mechanical Signals in Reactive Astrocytes
Source: Front Cell Dev Biol. 2021 Aug 23;9:712627. doi: 10.3389/fcell.2021.712627 (PMC8419233; doi:10.3389/fcell.2021.712627)

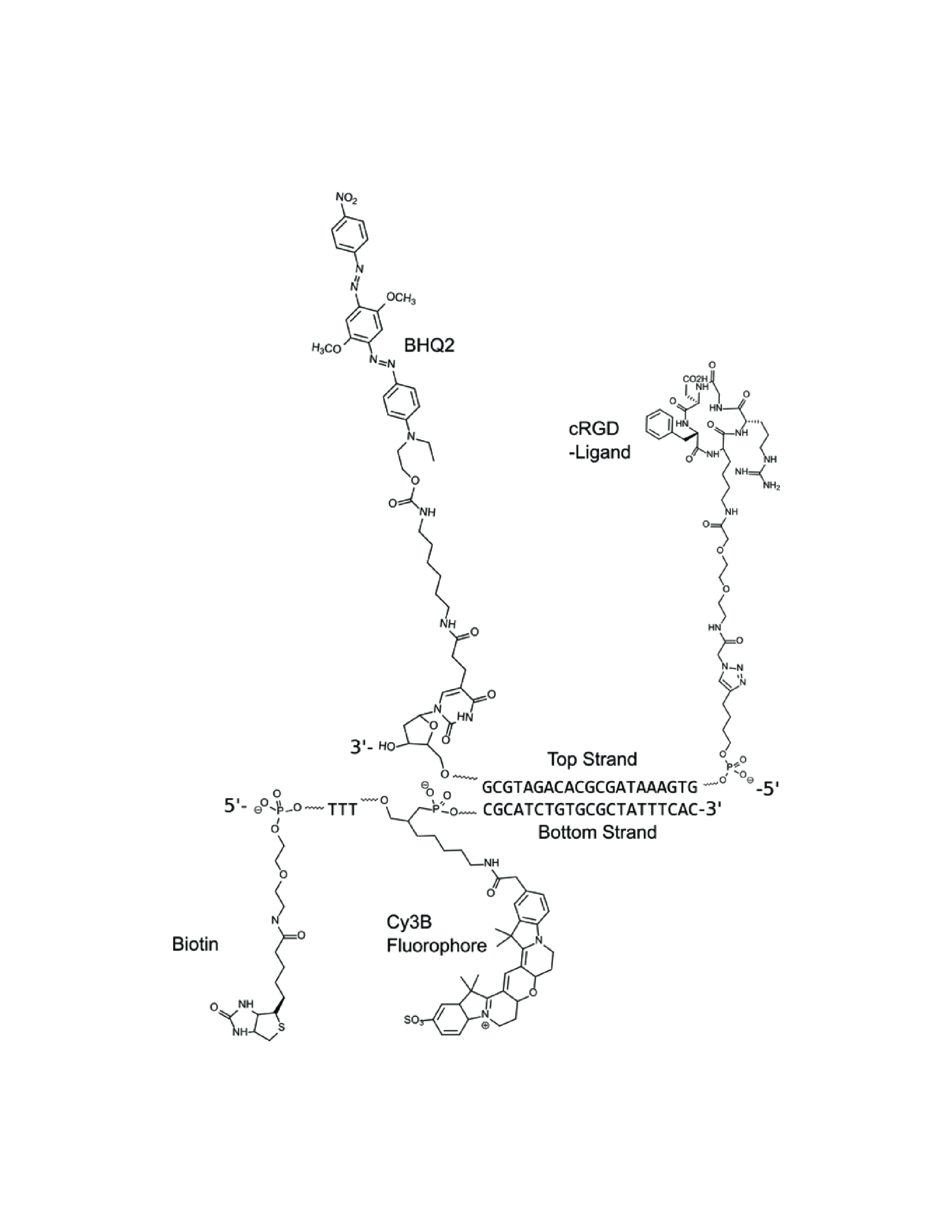

Supplement: Supplementary file 2 [file Image_1.JPEG]

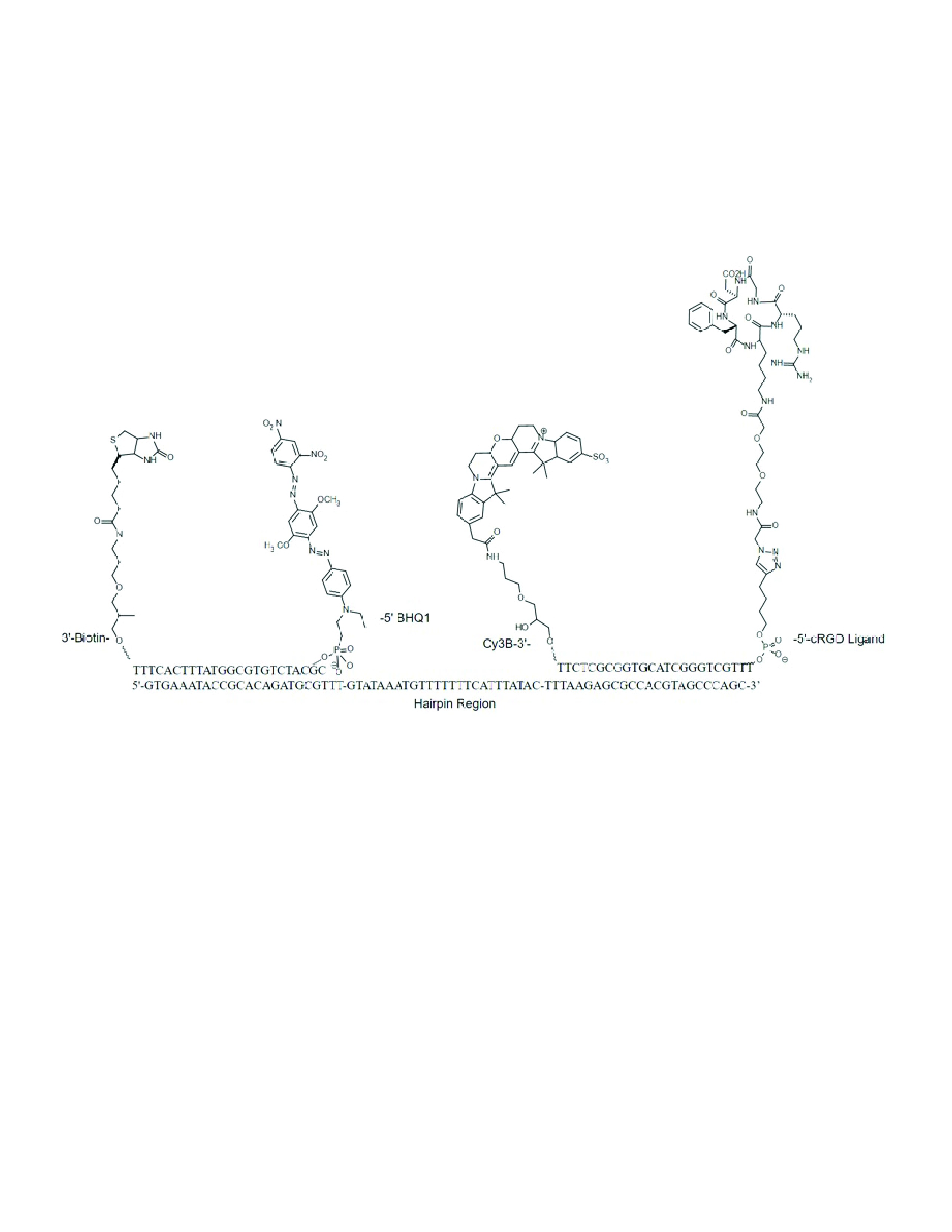

Supplement: Supplementary file 3 [file Image_2.JPEG]

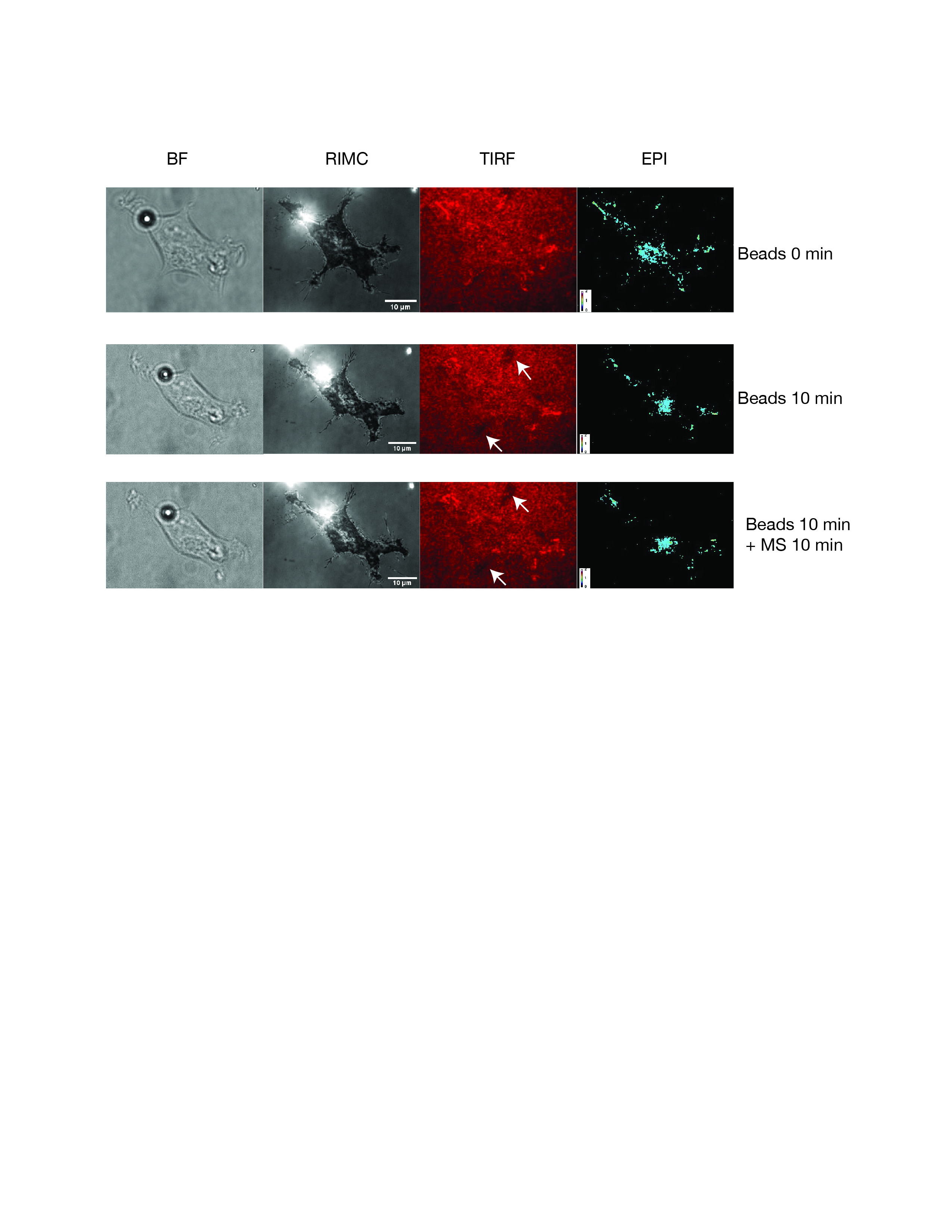

Supplement: Supplementary file 4 [file Image_3.JPEG]

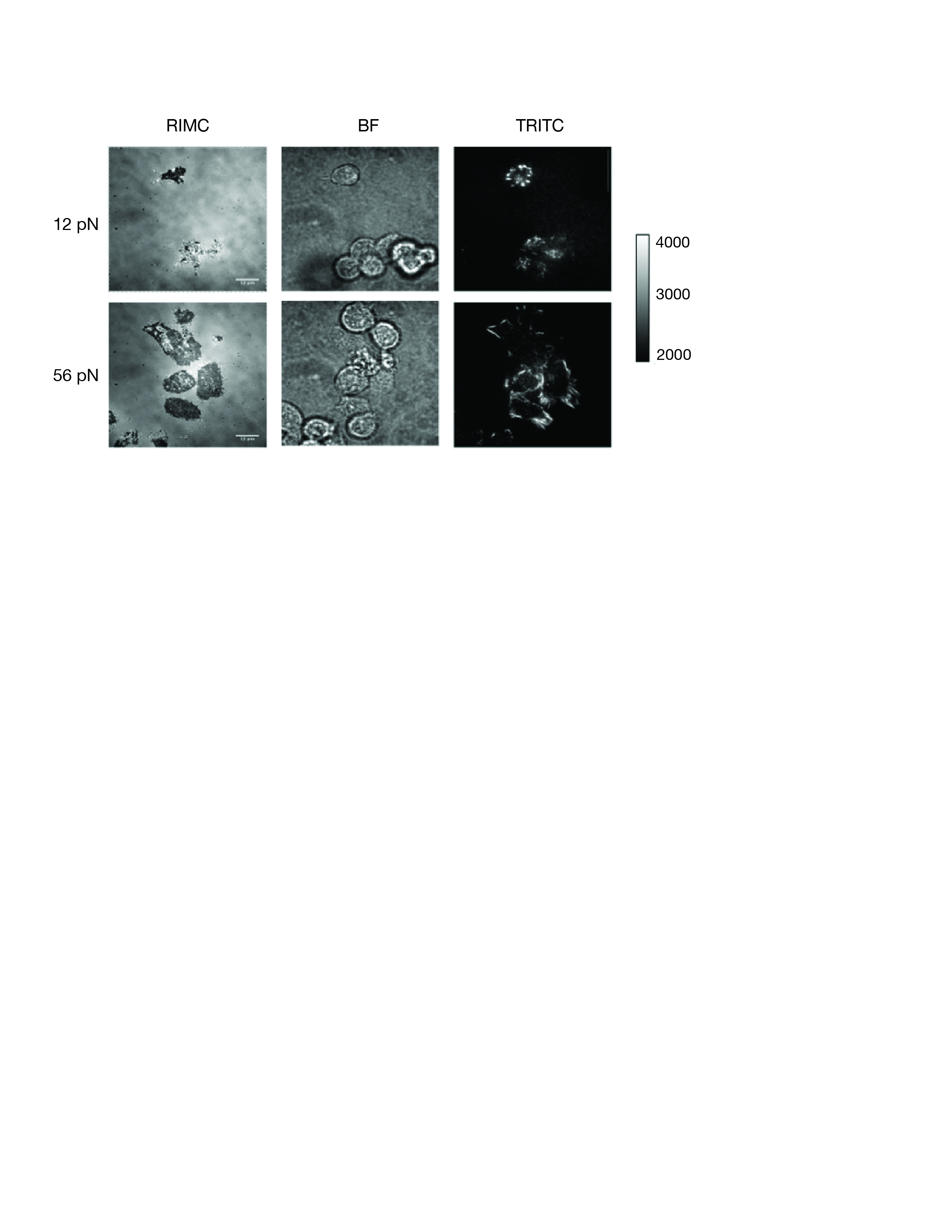

Supplement: Supplementary file 5 [file Image_4.JPEG]
